# Comprehensive characterization of potato *TBL* genes reveals candidates for salt and drought stress tolerance

**DOI:** 10.3389/fpls.2025.1741231

**Published:** 2026-01-26

**Authors:** Chenqi Wang, Xiaofeng Zhou, Bo Wang, Jianying Qiao, Zhiyong Xiong, Lei Wu

**Affiliations:** 1 Key Laboratory of Herbage and Endemic Crop Biology, Ministry of Education, Inner Mongolia University, Hohhot, Inner Mongolia, China; 2 College of Life Science, Inner Mongolia University, Hohhot, Inner Mongolia, China

**Keywords:** expression analysis, genome-wide, potato, stress response, *StTBLs*

## Abstract

The *Trichome Birefringence-Like* (*TBL*) gene family encodes polysaccharide acetyltransferases that modify polysaccharide properties, playing key roles in trichome development, cell wall acetylation, and responses to biotic and abiotic stresses. Potato, a globally important crop, frequently faces salinity and drought stress. However, the role of the potato *TBL* gene family in stress resistance remains unexplored. Using bioinformatics, we identified 72 St*TBL* genes in the potato DM1-3–516 R44 genome, unevenly distributed across 12 chromosomes. Phylogenetic analysis grouped them into three subfamilies with conserved domains including PC-Esterase, PMR5N, and DUF4283. We further examined gene structure, promoter cis-elements, predicted miRNA targets, GO annotations, and tissue-specific expression. Under both salt and drought stress, we identified several responsive candidate genes from the 72 *StTBLs*: 10 potential salt-responsive candidates (*StTBL 1*, *StTBL 3*, *StTBL 16*, *StTBL 20*, *StTBL 22*, *StTBL28*, *StTBL 58*, StTBL 59, *StTBL 60* and *StTBL 68*) and 11 potential drought-responsive candidates (*StTBL 1*, *StTBL 2*, *StTBL 3*, *StTBL 12*, *StTBL 19*, *StTBL 21*, *StTBL 22*, *StTBL 28*, *StTBL31*, *StTBL 33* and *StTBL 69*). This study presents the first genome-wide characterization of the *TBL* gene family in potato. The findings highlight candidate genes for improving salt and drought tolerance, offering insights for developing stress-resilient potato.

## 1 Introduction

Potato (*Solanum tuberosum* L.), a member of the Solanaceae family originating from the Andes of Peru and Bolivia (Spooner et al., 2005; Tang et al., 2022), is the world’s third largest food crop and a key contributor to global food security and agricultural economies. However, its production is severely constrained by soil salinization and recurrent drought. Climate change is making the weather more extreme, with problems of high temperatures, droughts, and high soil salinity becoming more frequent. Globally, salinity affects about 950 million hectares (about 7% of arable land) (Munns and Tester, 2008; Yang and Guo, 2018).

Traditional agronomic measures, such as applying organic fertilizer, water-saving irrigation, soil amendments, and grafting salt-tolerant rootstock, can partially mitigate these stresses but are costly, resource-intensive, and environmentally unsustainable. Therefore, the use of bioinformatics and molecular biology techniques to screen stress-resistant genes to provide theoretical targets for genetic improvement is an important channel for cultivating a new generation of super crops to cope with climate change.

The plant cell wall, composed mainly of cellulose, hemicellulose, and lignin, is essential for cell morphology, defense, signal transduction, and development (Keegstra, 2010). Xylan, interconnected with cellulose and lignin, forms a complex network that strengthens the cell wall (Wen et al., 2024). Its acetylation is vital for proper wall assembly and mechanical stability (Yuan et al., 2016a). The *Trichome Birefringence-Like* (*TBL*) family encodes polysaccharide O-acetyltransferases involved in the acetylation of xylan (Lunin et al., 2020; Urbanowicz et al., 2014; Zhong et al., 2017), xyloglucan (Zhong et al., 2018a, Zhong et al., 2020), mannan (Zhong et al., 2018b), and pectin (Stranne et al., 2018). The *TBL* gene family is mainly involved in the modification of cell wall polysaccharides via acetyltransferase activity. TBL3 (Bischoff et al., 2010b; Yuan et al., 2016c), TBL22/AXY4L (Gille et al., 2011), TBL27/AXY4 (Gille et al., 2011), and TBL28-35 (Yuan et al., 2016b) in Arabidopsis belong to structural proteins. These proteins have acetyltransferase activity and dynamically regulate cell wall properties through chemical modification. In *A. thaliana*, xylan *O*-acetyltransferase 1 (XOAT1) specifically acetylates the xylan backbone (Lunin et al., 2020), while DUF231 proteins (e.g., TBL3, TBL28, TBL29/ESK1, TBL30–35) utilize acetyl-CoA to acetylate xylooligomer (Grantham et al., 2017). Loss of *TBL3* decreases crystalline secondary cell wall cellulose in trichomes and stems (Bischoff et al., 2010a), underscoring the family’s pivotal role in cell wall biosynthesis.

*TBL* genes also influence stress responses (Bischoff et al., 2010a; Xin et al., 2007) and disease resistance (Bi et al., 2024; Chen et al., 2020; Gao et al., 2017; Wen et al., 2025; Zhang et al., 2025a). In *A. thaliana*, the acetylation of xylan mediated by *TBL29* (*ESK1*) is a necessary step to maintain the structural integrity and mechanical support function of the catheter. The *esk1* mutant enhances frost tolerance without acclimation (Xin et al., 2007), acting as a negative regulator, while *tbl44* shows resistance to powdery mildew (Chen et al., 2020), and *tbl27* is hypersensitive to aluminum stress (Gao et al., 2017). In *Nicotiana tabacum*, *NtTBL31* contributes to drought tolerance (Wang et al., 2025). In rice, mutations in *OsTBL1* and *OsTBL2* reduce wall acetylation and increase susceptibility to leaf blight (Zhang et al., 2025a). In roses, *RcTBL16* mediates interactions with *Botrytis cinerea* (Kumar et al., 2016). The *tbl10* mutant in *A. thaliana* showed reduced RG-I acetylation and enhanced drought tolerance (Stranne et al., 2018). Collectively, these findings suggest that *TBL* genes play diverse roles in stress adaptation and pathogen defense. The above AtTBL29, AtTBL44, OsTBL1, OsTBL2 and RcTBL16 are regulatory TBL proteins.

Because cell walls are the first barrier against environmental stress (Bi et al., 2024; Zhang et al., 2025b), their composition and modification are central to plant resilience. Despite this significance, the *TBL* gene family has not been systematically chara-cterized in potatoes. To address this gap, we performed a genome-wide identification and analysis of *stTBLs*, including gene structure, phylogeny, cis-regulatory elements, GO annotation, and stress-responsive expression in the Atlantic tetraploid variety under salt (200 mM NaCl) and drought (200 mM mannitol) stress for 0–96 h. Several candidate genes associated with salt and drought tolerance were identified. This study revealed the role of *TBL*-mediated o-acetylation in potato stress response, screened potential salt- responsive and drought-responsive candidates, and provided targets and directions for molecular breeding.

## 2 Materials and methods

### 2.1 Identification of *StTBL* genes, physicochemical properties, chromosome localization, and phylogenetic analyses

The complete genome and protein sequences of the potato DM variety were obtained from the SpudDB database (https://spuddb.uga.edu/dm_v6_1_download.shtml, accessed 4 January 2025). Candidate *StTBL* genes were identified using the HMM profile for the PC-Esterase (PF 13839), with protein domain downloaded from the Pfam database (https://pfam.xfam.org/, accessed 30 October 2024). Physicoche-mical properties of the encoded membrane proteins were analyzed with TBtools v2.210 (Chen et al., 2020), and subcellular localization was predicted using Cell-PLoc 2.0 (http://www.csbio.sjtu.edu.cn/bioinf/Cell-PLoc-2/, accessed 24 April 2024). Genes’ positions on chromosomes were visualized with TBtools, and members of the *StTBL* family were renamed according to their chromosomal order.

Protein sequences of *Arabidopsis thaliana* were retrieved from TAIR (https://www.arabidopsis.org/, accessed 2 June 2024) and extracted using the Fasta Extract Filter (Quick) function in Tbtools v2.210 (Bischoff et al., 2010a; Chen et al., 2020; Gao et al., 2017). Multiple sequence alignments of potato and Arabidopsis TBL proteins were performed with ClustalW using default parameters. Phylogenetic analysis of the *StTBL* gene family was conducted in MEGA 7.0 software (Kumar et al., 2016) using the maximum likelihood (ML) method with 1,000 bootstrap replicates and default settings. The resulting phylogenetic tree was visualized and refined with Evolview (https://www.evolgenius.info/evolview/#/treeview, accessed 6 June 2025).

### 2.2 Analysis of conserved motifs, domains, and gene structure of *StTBL* genes

Exon-intron structures of *StTBL* genes were analyzed by aligning cDNA sequences with their corresponding genomic DNA sequences. Conserved motifs were identified using MEME (Multiple Em for Motif Elicitation; https://meme-suite.org/meme/tools/meme, accessed 28 April 2025) (Bailey et al., 2006), with the maximum number of motifs set to 10 and other parameters left at default. Conserved domains were annotated using the NCBI CDD database (https://www.ncbi.nlm.nih.gov/Structure/cdd/wrpsb.cgi, accessed 28 April 2025). Gene structures were analyzed and visualized with TBtools v2.210 (Chen et al., 2020) using GTF/GFF3 files. Finally, the phylogenetic tree, conserved motifs, and gene structures were integrated using the Gene Structure View (Advanced) function in TBtools v2.210 (Chen et al., 2020).

### 2.3 Analysis of gene duplication, collinearity, and the ratio of nonsynonymous (Ka) to synonymous (Ks) nucleotide substitutions

Genome, genome annotation, and CDS sequence files of *Solanum lycopersicum*, *A. thaliana*, *O. sativa*, *Triticum aestivum*, and *Zea mays* were downloaded from Ensemblplants (https://plants.ensembl.org/index.html, accessed 11 May 2025). The ge-nome and annotation data of *Nicotiana benthamiana* were obtained from the Sol Genomics Network (https://solgenomics.net/, accessed 11 May 2025). CDS sequences of *N. benthamiana* were extracted from the genome and annotation files using Linux commands.

Gene duplication and collinearity analyses of the *TBL* gene family were performed using the Advanced Cricos plug-in in TBtools v2.210 (Chen et al., 2020). Interspecies collinearity of *TBL* genes of *S. tuberosum* with *S. lycopersicum*, *N. benthamiana*, *A. thaliana*, *O. sativa*, *T. aestivum*, and *Z. mays* was analyzed using TBtools v2.210 (Chen et al., 2020) and MCScanX (http://chibba.pgml.uga.edu/mcscan2/, accessed 11 May 2025) (Wang et al., 2012). The Ka and Ks substitution rates of duplicated gene pairs were calculated using TBtools v2.210 (E-value cut-off < 1 × 10^-10^ and num of BlastHits with 5) (Chen et al., 2020; Yuan et al., 2024), and the Ka/Ks ratio was used to infer the evolutionary patterns of *StTBL* genes. Ka/Ks values within and between potato species were plotted using OriginPro 2024 (OriginLab Corporation, Northampton, MA, USA).

### 2.4 Analysis of cis-acting elements in the promoter region of *StTBL* genes

The 2000 bp upstream promoter sequences of potato *StTBL* coding regions were extracted using TBtools v2.210 (Chen et al., 2020). Cis-regulatory elements related to plant growth and development, hormone response, and stress response were predicted using the PlantCARE database (https://bioinformatics.psb.ugent.be/webtools/plantcare/html/, accessed 15 April 2025). Elements lacking clear annotation or biological relevance were excluded. The distribution of cis-acting elements was visualized as heatmaps using TBtools v2.210 (Chen et al., 2020).

### 2.5 Gene ontology annotation and miRNA target prediction of St*TBL* genes

The Gene Ontology (GO) enrichment file (DM_1-3_516_R44_potato.v6.1. working_models.go.txt) was downloaded from SpudDB (https://spuddb.uga.edu/dm_v6_1_download.shtml, accessed 21 May 2025). GO enrichment analysis was performed using the clusterProfiler package in R, and results were ranked by enrichment factor (Yu et al., 2012).

CDS of 72 *StTBLs* were extracted from the SpudDB file (DM_1-3_516_R44_ potato.v6.1.working_models.cds.fa, https://spuddb.uga.edu/dm_v6_1_download.shtml, accessed 4 January 2025) using the seqkit command in Linux. Potential miRNAs targeting *StTBL* genes were predicted with psRNATarget (https://www.zhaolab.org/ps-RNATarget/, accessed 18 May 2025) using default parameters (The expectation is set to 5). Interaction networks between miRNAs and *StTBL* genes were visualized with Cytoscape 3.9.1.

### 2.6 Expression analysis of *StTBL* genes in different tissues

The expression patterns of *StTBL* genes in various tissues of DM potato were comprehensively analyzed using RNA-seq data retrieved from the PGSC database (https://spuddb.uga.edu/dm_v6_1_download.shtml, accessed on 4 January 2025). Ba-sed on the publicly available transcriptome data of potato tissues, the expression status of potato *TBL* family genes in different tissues and organs was studied by FPKM (Fragments Per Kilobase of transcript per Million mapped reads) value analysis method. The log2 (FPKM + 1) formula is used for calculation and the heat map is drawn. Raw sequences are available in the National Center for Biotechnology Information Sequence Read Archive under BioProject PRJNA753086 (Brose et al., 2025).

### 2.7 Expression of *StTBL* genes under drought and salt stress

Stem segments of potato (*Solanum tuberosum* L. cv. Atlantic) were cultured on MS solid medium (TQ-AL-PL361, Techisun, Shenzhen, China) for 35 days under controlled conditions (22 ± 2°C, light intensity 25.0-37.5 μmol m^-2^ s^-1^, 16 h light/8 h dark). MS solid medium containing 200 mM NaCl (4.41 g MS solid medium and 11.688 g NaCl (HDM-7647-14-5A5, TIANJINKEMAO, Tianjin, China) were added to 1L of distilled water), MS solid medium containing 200 mM mannitol (4.41 g MS basic medium and 36.4 g mannitol (CM7091-500g, Coolaber, Beijing, China) were added to 1L of distilled water) and ordinary MS solid medium (4.41 g solid medium was added to 1L of distilled water) were prepared (Garner and Blake, 1989; Zhu et al., 2020). The pH was adjusted to 5.8 and sub-packed in a test tube with a bottom diameter of 2.4 cm and a height of 18 cm. The height of the solid medium was 5 cm. High pressure sterilization 121°C 15min. The potato tissue culture seedlings with consistent growth were selected, and the roots were rinsed with sterile water, dried with filter paper, and transferred to MS test tube medium containing 200 mM mannitol or 200 mM NaCl. The potato tissue culture seedlings in the control group were transferred to ordinary MS solid medium. Drought stress was simulated using 200 mM mannitol. Nine replicate samples were set for each of the six groups (0, 12, 24, 36, 72 and 96 h). Samples were collected at 0,12,24,36,72 and 96 h after the start of stress. Three potato tissue culture seedlings with consistent growth were selected, and their leaf tissues were taken, frozen in liquid nitrogen and stored at − 80°C for further analysis. This experiment was repeated three times. Atlantic was gifted by the Inner Mongolia Potato Virus-free Seed Potato Breeding Center, China.

### 2.8 RNA extraction and quantitative real-time PCR (qRT-PCR) analysis

Total RNA was extracted from potato leaves using standard protocols. First-strand cDNA was synthesized with the All-in-One First-Strand Synthesis MasterMix (with dsDNase) for qPCR (Cat#EG15133S, BestEnzymes, Nanjing, Jiangsu, China). Primers were designed using SnapGene software (https://www.snapgene.com). qRT-PCR was performed on a QuantStudio^®^ 3 Real-Time PCR Instrument (96-well 0.2 ml Block; Cat#A28567, Thermo Fisher Scientific, USA) using F488 SYBR qPCR Mix (Universal) (Cat#EG23111L, BestEnzymes, Nanjing, Jiangsu, China). The housekeeping gene *StTubulin* (*PGSC0003DMC400020469*) served as the internal control. Amplification conditions were 95°C for 30 s followed by 40 cycles of 95°C for 10 s, and 60°C for 30 s. Relative expression levels of target genes were calculated using the 2^-ΔΔCt^ method. Ct values were obtained from four biological replicates, with two technical replicates. The average relative expression of each group of genes was used to draw the heat map of cluster analysis using the HeatMap plug-in of TBtools v2.210 (Chen et al., 2020). The standard curves of cDNA (1, 2, 4, 10,10^2^,10^3^,10^4^,10^5^ × dilutions) diluted in a series of control groups were constructed, and the amplification efficiency of each pair of primers was calculated. The formula was E (%) = [10 ^(− 1/slope)^ − 1] × 100. The average cycle threshold (Ct) of each gene was obtained by four biological repeats, and each biological repeat was composed of two technical repeats (Fan et al., 2022; Livak and Schmittgen, 2001; Radonic et al., 2004). The primer sequences and amplification efficiency of 72 *StTBL* genes and reference genes were shown in **Supplementary Table S8**. The result of qRT-PCR melting curve analysis showed that there was a single smooth curve with a TM of the target gene around 80°C (**Supplementary Figure S1**).

### 2.9 Homology analysis of StTBL and AtTBL proteins

Through the BLAST plug-in of TBtools v2.210 (Chen et al., 2020), the sequences of 72 StTBL proteins and 46 AtTBL proteins were compared. Plotting using DNAMAN (version 8.0, Lynnon Biosoft, Quebec, Canada).

## 3 Results

### 3.1 Identification, physicochemical properties, phylogenetic analysis, and chromosomal distribution of StTBL proteins

**Figure 1:**
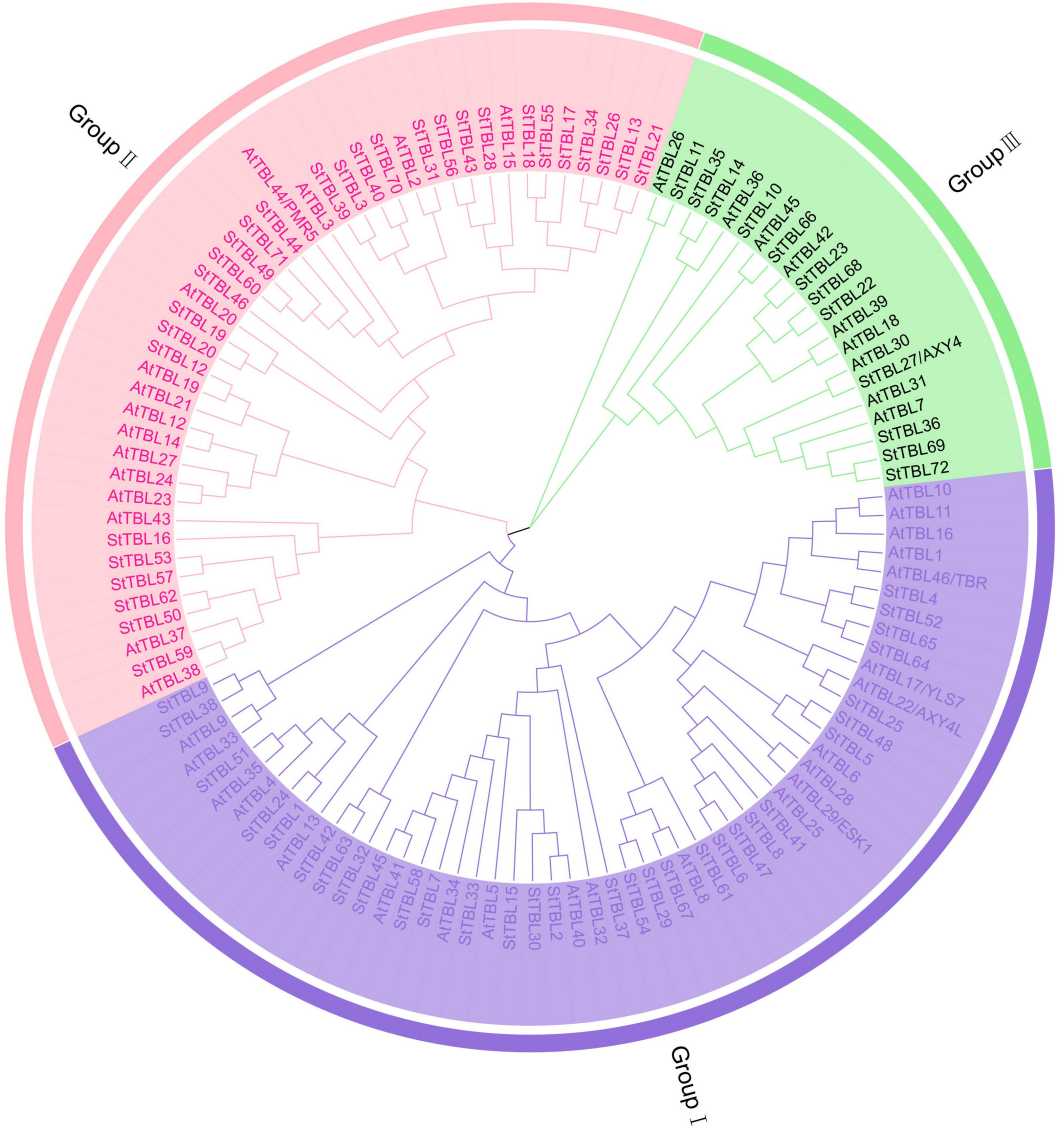
Phylogenetic analysis of potato and Arabidopsis TBL proteins resolved 72 StTBLs and 46 AtTBLs into three groups (I-III), with distinct color-coding demarcating cladistic affiliations. The maximum likelihood phylogeny was constructed using MEGA 7, implementing 1000 bootstrap replicates based on TBL amino acid sequence alignments.

Based on the potato DM genome, 102 candidate StTBL proteins were initially identified using the Hidden Markov Model (HMM) profile of the PC-Esterase domain (PF13839). After domain validation with SMART and Pfam, redundant sequences (When the amino acid sequence similarity of the protein encoded by the gene is > 90% and the coverage length is > 90%, it is judged to be a redundant gene.) were removed, yielding 72 StTBL proteins (**Figure 1**, **Supplementary Table S1**). Phylogenetic analysis was conducted using 72 StTBL and 46 AtTBL protein sequences **Supplementary Table S2**. The neighbor-joining tree divided the proteins into three groups: Group I (31 potato, 22 Arabidopsis), Group II (29 potato, 15 Arabidopsis), and Group III (12 potato, 9 Arabidopsis). Detailed protein sequence data are provided in **Supplementary Table S2**.

**Figure 2:**
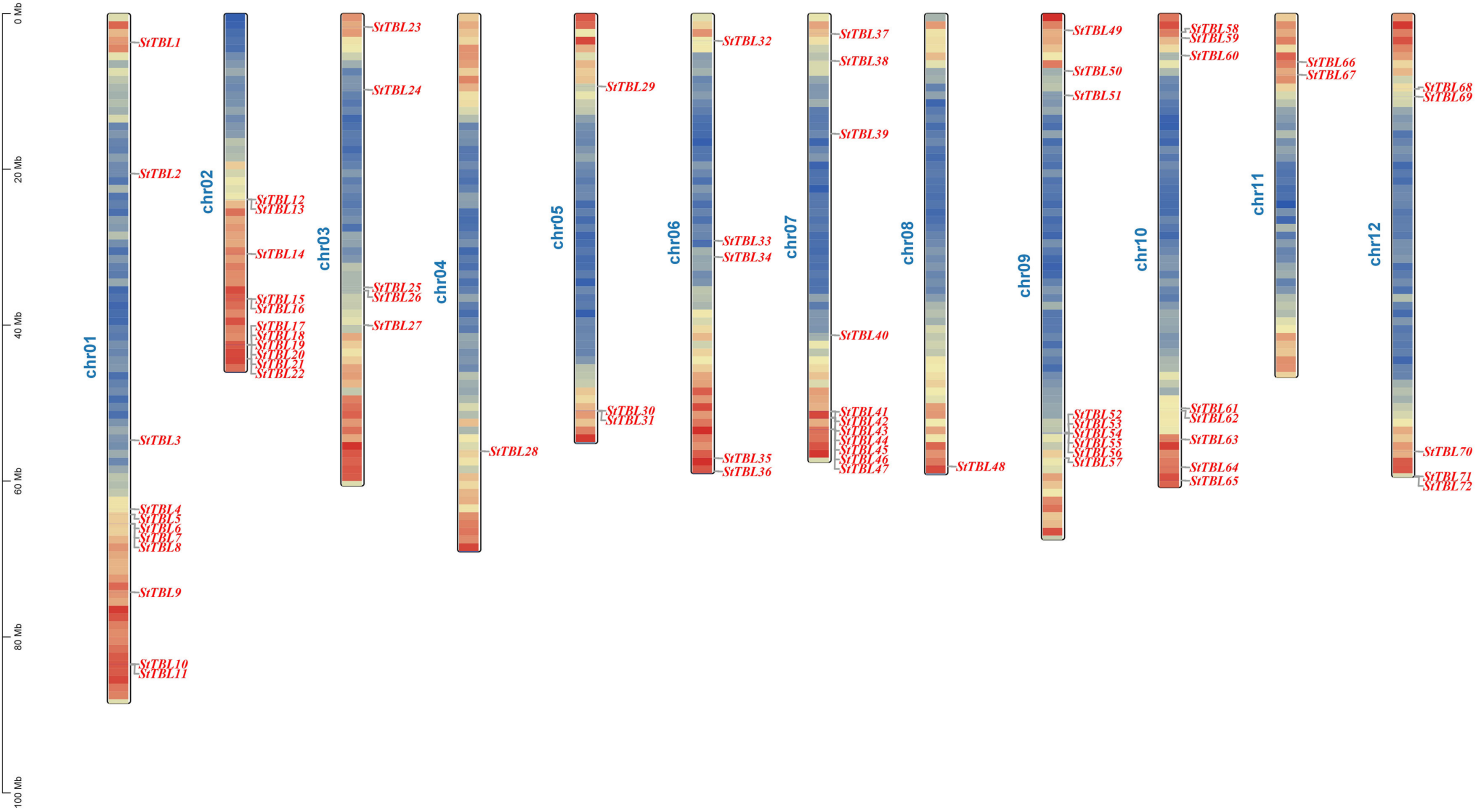
Chromosome distribution of *StTBL* gene family members. The colored rectangular bars represent the chromosomes of DM potatoes, marking the number and length of chromosomes (Mb).

Analysis of physicochemical properties of StTBL proteins revealed that StTBL proteins ranged from 98 (StTBL39) to 846 (StTBL30) amino acid residues, with molecular weights from 10727.21 Da (StTBL39) to 98974.08 Da (StTBL30). Theore-tical isoelectric points (pI) ranged from 5.43 (StTBL7) to 10.04 (StTBL43), with 67% of proteins being basic (pI > 7.0). Instability index varied from 20.15 (StTBL45) to 59.8 (StTBL25), with 41 proteins classified as stable (index < 40). The aliphatic index ranged from 62.14 (StTBL65) to 98.56 (StTBL7). All proteins were hydrophilic, with GRAVY values ranging from –0.742 (StTBL21) to –0.107 (StTBL67). Subcellular localization predicted 24 proteins in the plasma membrane, 8 in the cell wall, 33 in the chloroplast, 1 in the cytoplasm, 1 in the mitochondria, and 5 in the nucleus (**Supplementary Table S1**). Chromosomal mapping showed that the 72 *StTBL* genes were unevenly distributed across 12 chromosomes (**Figure 2**). Chr01, Chr02, and Chr07 each contained 11 genes, while Chr04 and Chr08 had only one gene each. Genes were named *StTBL1*–*StTBL72* according to their chromosomal order (**Supplementary Table S1**).

### 3.2 Conserved motifs, domains, and gene structures of *StTBL* genes

**Figure 3:**
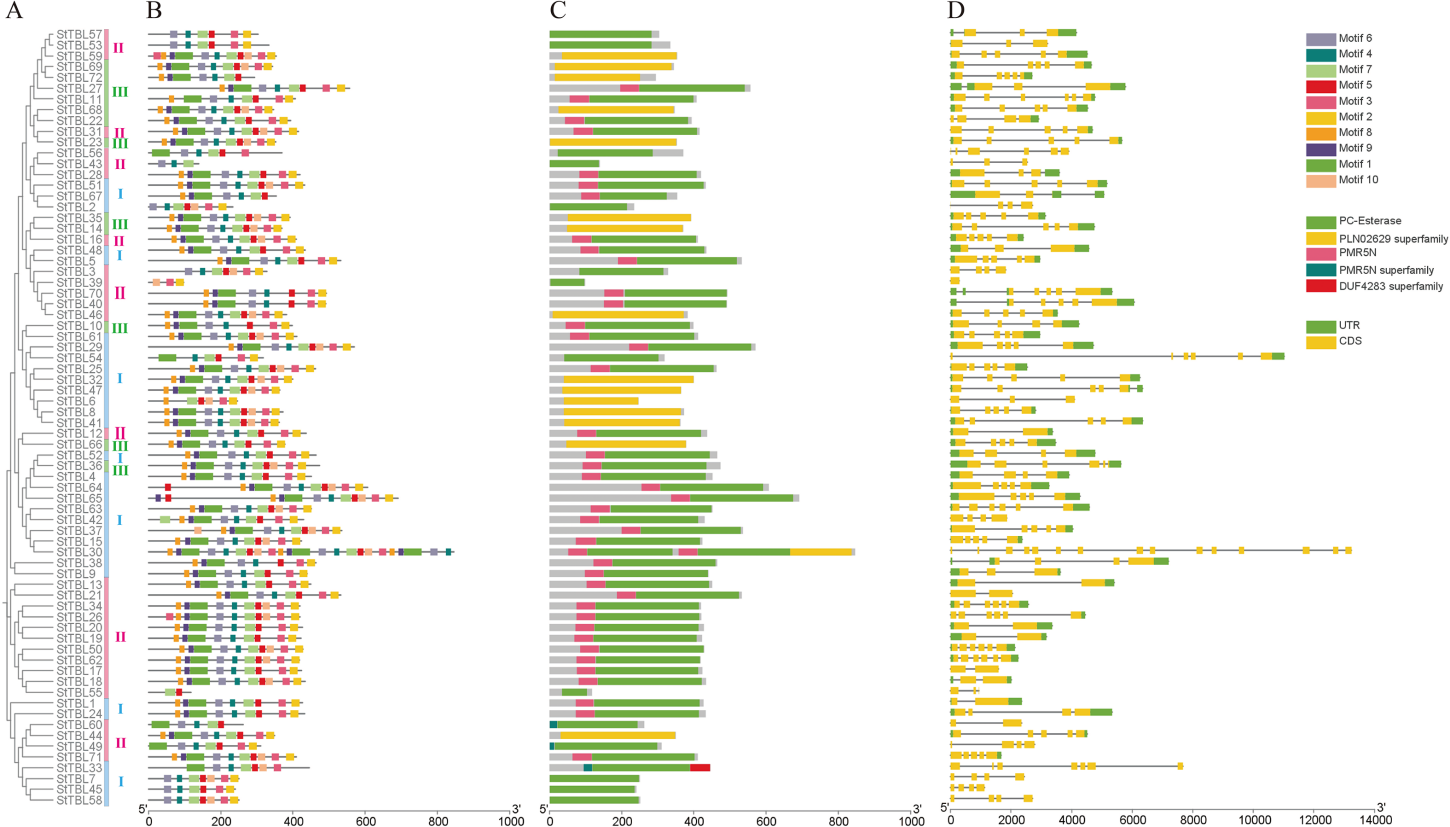
Phylogenetic relationship, conserved motifs, and gene structure of *StTBLs*. **(A)** A phylogenetic tree was constructed based on the full-length sequences of 72 potato TBL family proteins. Color-coding: Group I (blue), Group II (pink), Group III (green). **(B)** The conserved motif of the StTBL protein. The gray horizontal line represents the amino acid length of the sequence, and the different colors on the sequence represent various motif types. **(C)** StTBLs protein domain. The colored part marks different conserved domains, while the gray area indicates an area without a specific domain. **(D)** The way the *StTBL* genes are put together, with the exon and intron parts. UTR, untranslated region. The gray horizontal line represents the intron region, the green corresponds to the UTR, and the yellow corresponds to the CDS region.

Ten conserved motifs (motifs 1 − 10) were identified using the MEME program (**Supplementary Figure 2**). The predominant motif arrangement was motif 8 → motif 9 → motif 1 (GCD) → motif 6 → motif 4 → motif 7 → motif 5 → motif 10 → motif 3 → motif 2 (Asp-X-X-histidine, DXXH). Notably, Group III contained both the GCD and motif 4 (**Figures 3A, B**). The amino acid sequences of GCD, motif 4, and DXXH were DYLKWRWQPNDCELPRFBAKQFLEKQFLEKLRGKRJMFVGDSLNRNQ WZSLVCLL, WKGADVLIFNTGHWWW, and QDCSHWCLPGVPDTWNELLYAL L, respectively (**Supplementary Figure 2**). Conserved domain analysis showed that 57 genes carried a PC-esterase domain, and 43 of these also contained a PMR5N domain. Group I included 26 PC-esterase and 20 PMR5N domain genes; Group II comprised 26 PC-esterase and 16 PMR5N domain genes; and Group III contained 5 PC-esterase and 6 PMR5N domain genes. *StTBL30* had the highest exon count, 15, whereas *StTBL39* contained only one exon. Most *StTBL* genes carried 3 − 6 exons (**Figure 3D**).

### 3.3 Duplication and collinearity analysis of *StTBL* genes between potato and six other plants

**Figure 4:**
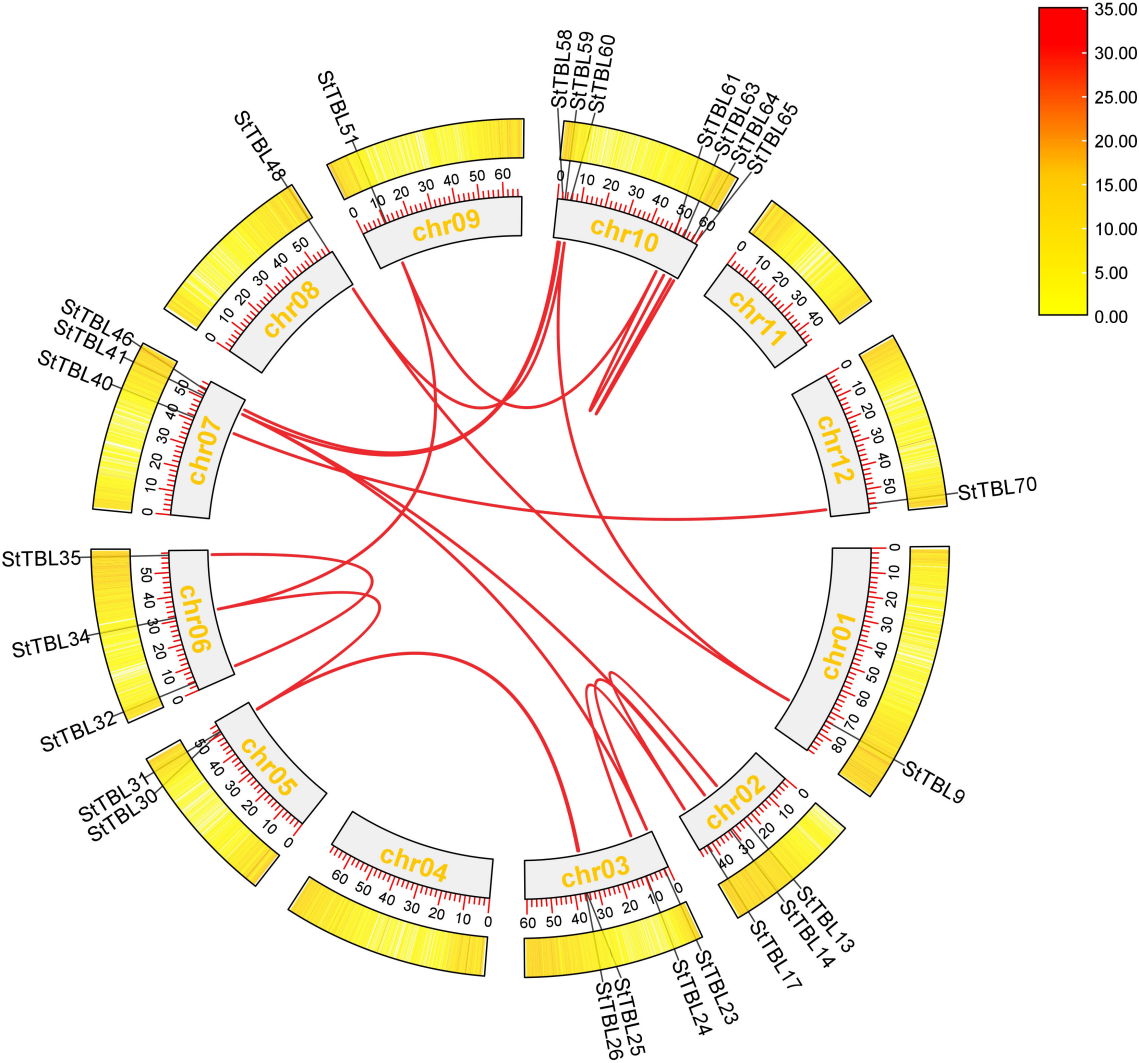
The synthetic relationship of the *StTBL* gene in potatoes was shown. The gray circle inside represents the potato chromosome, and the position of the *StTBL* gene is displayed on the circle. The internal white band delineates the syntenic region blocks in the potato genome, whereas the red band denotes segmental duplication events.

**Figure 5:**
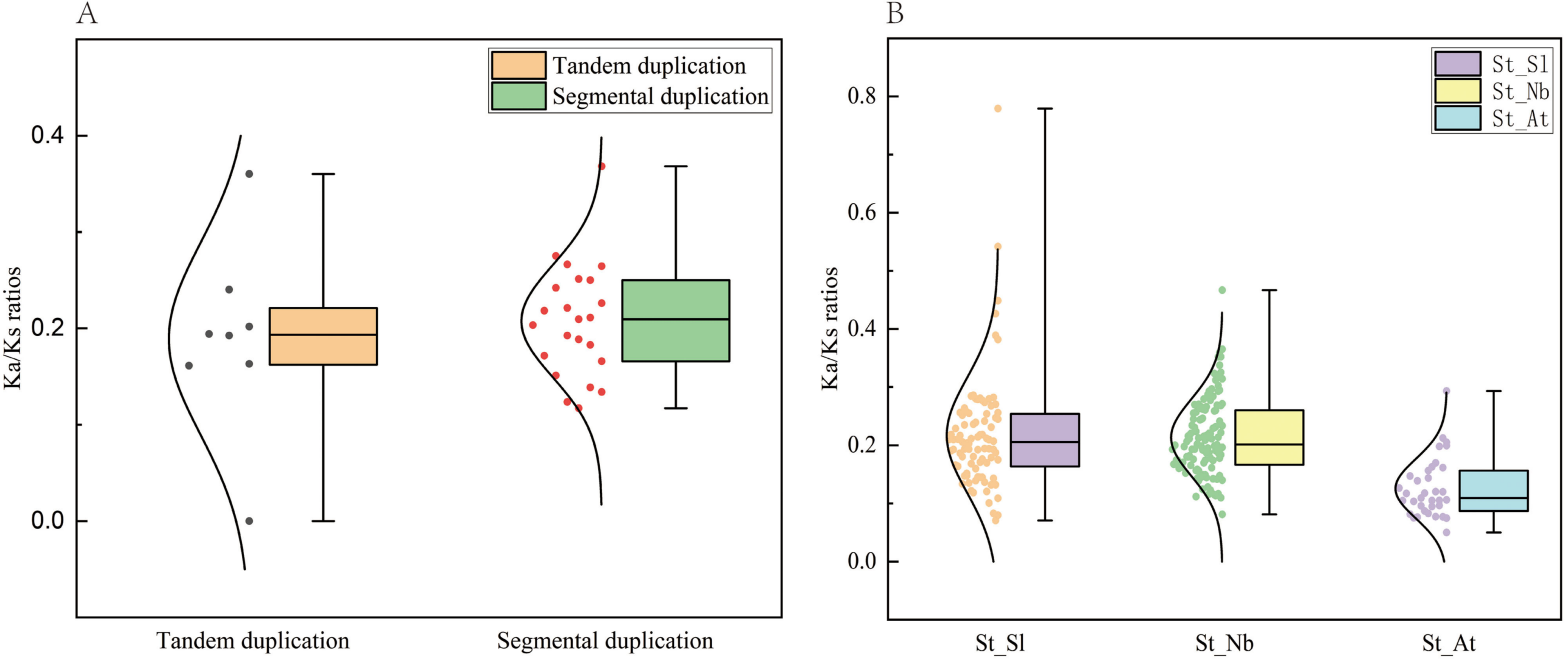
Ka/Ks values of *TBL* genes within potato species (tandem repeats and segmental repeats) and between species (*S. tuberosum* and *S. lycopersicum*, *S. tuberosum* and *N. benthamiana*, *S. lycopersicum* and *A. thaliana*).

**Figure 6:**
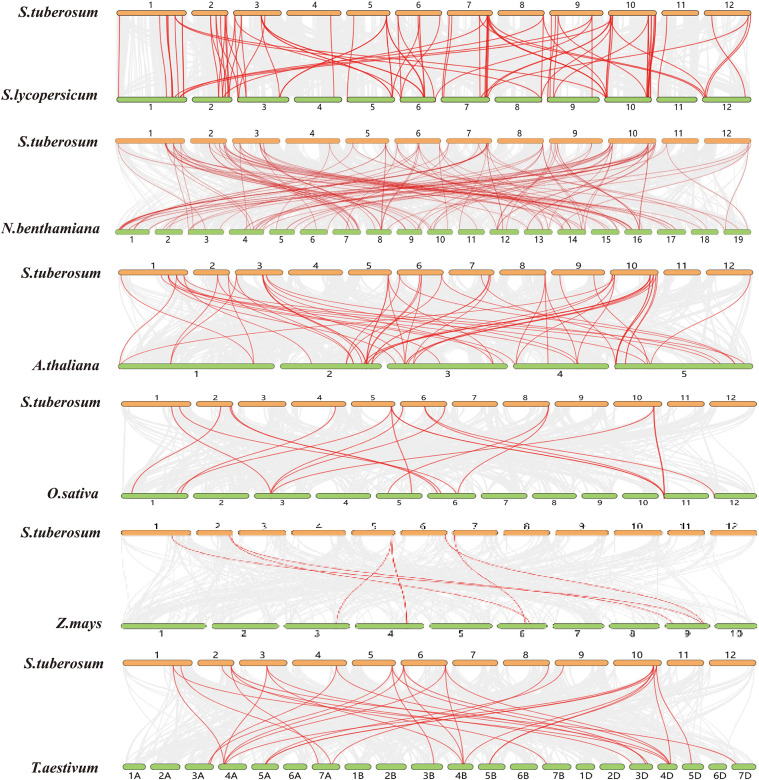
**A** comparison of *TBL* genes between *S. tuberosum* and six other plants was performed using identical linear analysis. The grey line represents the gene blocks orthogonal to other genomes in *S. tuberosum*. The red line depicts the same line *TBL* gene pair.

In potato, eight tandemly duplicated *StTBL* gene pairs and 23 segmental duplication events were detected across different chromosomes (**Figure 4**; **Supplementary Table S3**). These duplications likely contributed to the expansion of the *StTBL* family (**Figure 4**). We calculated Ka/Ks ratios of duplicate gene pairs, and all values were < 1 (**Figure 5**). Collinearity analysis was then conducted between potato and six representative species. We identified 91 orthologous pairs with *S. lycopersicum*, 126 with *N. benthamiana*, 62 with *A. thaliana*, 19 with *O. sativa*, 31 with *T. aestivum*, and 8 with *Z. mays* (**Figure 6**; **Supplementary Table S4**). Among these, 17 *StTBL* genes showed collinearity with 3–8 *N. benthamiana* genes. Similarly, 13 genes had collinearity with three *S. lycopersicum* genes, while 6 genes were collinear with 3–4 *A. thaliana* genes. Fewer relationships were observed with monocots: one potato gene was collinear with three *O. sativa* genes, and several had collinearity with *T. aestivum*. No potato gene exhibited collinearity with multiple *Z. mays* genes (**Supplementary Table S4**). Ka/Ks values for orthologous pairs between potato and *A. thaliana*, *S. tuberosum*, and *S. lycopersicum* were consistently < 1, with some cases showing Ka=0 or undefined Ka/Ks values, likely due to extreme evolutionary conservation. By contrast, comparisons with monocots (*O. sativa*, *T. aestivum*, and *Z. mays*) yielded undefined values, likely due to low sequence homology preventing reliable estimation of substitution rates (**Supplementary Table S4**).

### 3.4 Analysis of cis-acting elements in the *StTBL* genes’ promoters

**Figure 7:**
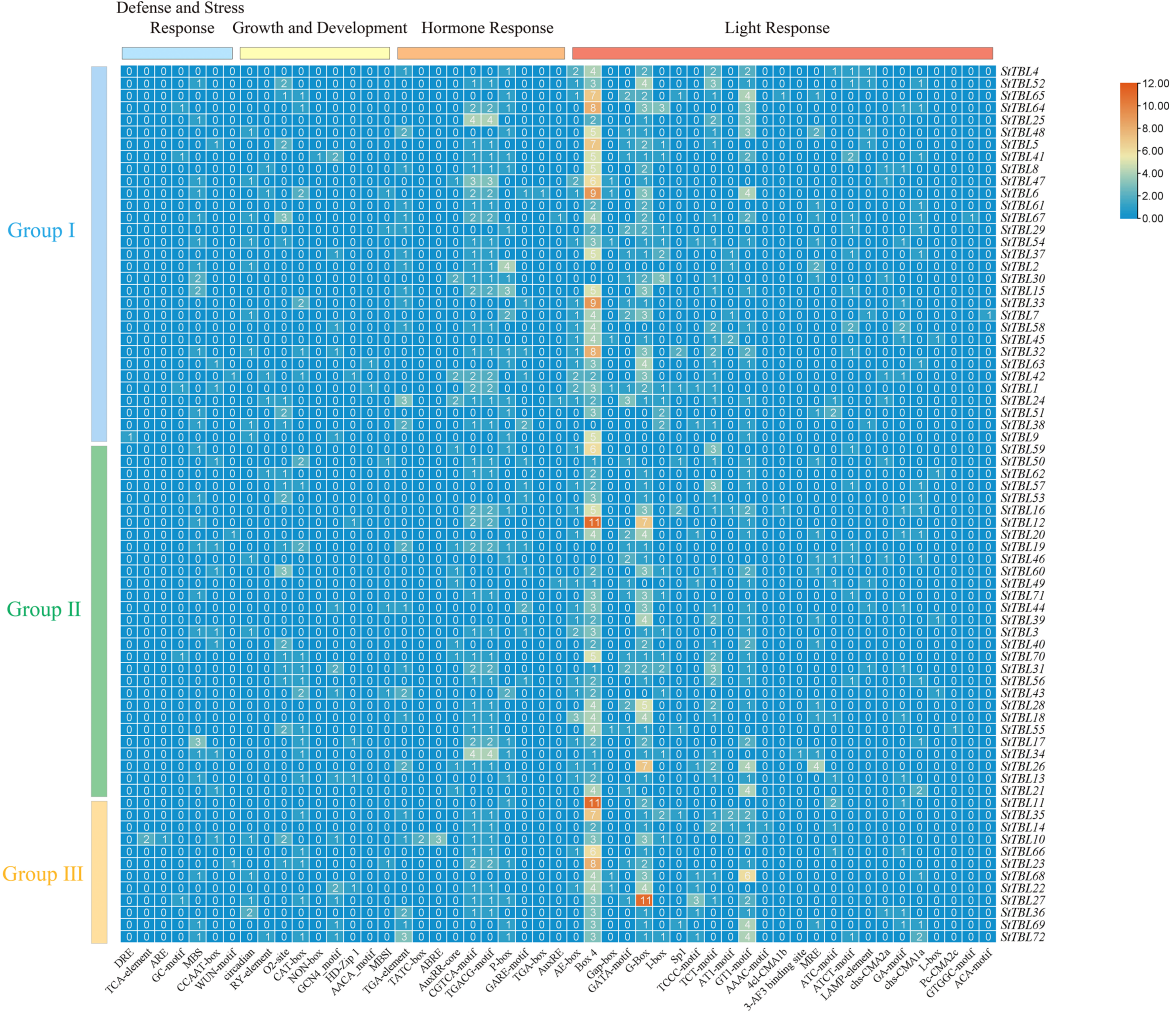
Cis-regulatory architecture of *StTBL* promoters.

The 2000 bp upstream sequences of *StTBL* genes were analyzed for cis-acting elements. A total of 1,218 elements were identified, grouped into four major categories: defense and stress response (51, 4.1%), growth and development (111, 9.1%), hormone response (239, 19.6%), and light response (817, 67.0%). Light-responsive elements were the most abundant, accounting for two-thirds of all elements. Among the defense and stress response category, drought-inducible elements were the most frequent (39, 3.2%), with MBS occurring 28 times; MBS is known to regulate drought-induced gene expression (Tan et al., 2025). Hormone-responsive elements included auxin response (53, 4.4%), gibberellin response (53, 4.4%), and MeJA response (130, 10.7%), with MeJA elements being most abundant. In the growth and development category, elements associated with zein metabolism regulation (31, 3.5%), meristem expression (28, 2.3%), endosperm expression (17, 1.4%), and circadian rhythm control (16, 1.3%) were relatively enriched (**Figure 7**; **Supplementary Table S5**).

### 3.5 Gene Ontology annotation and miRNA analysis of potato *TBL* genes

**Figure 8:**
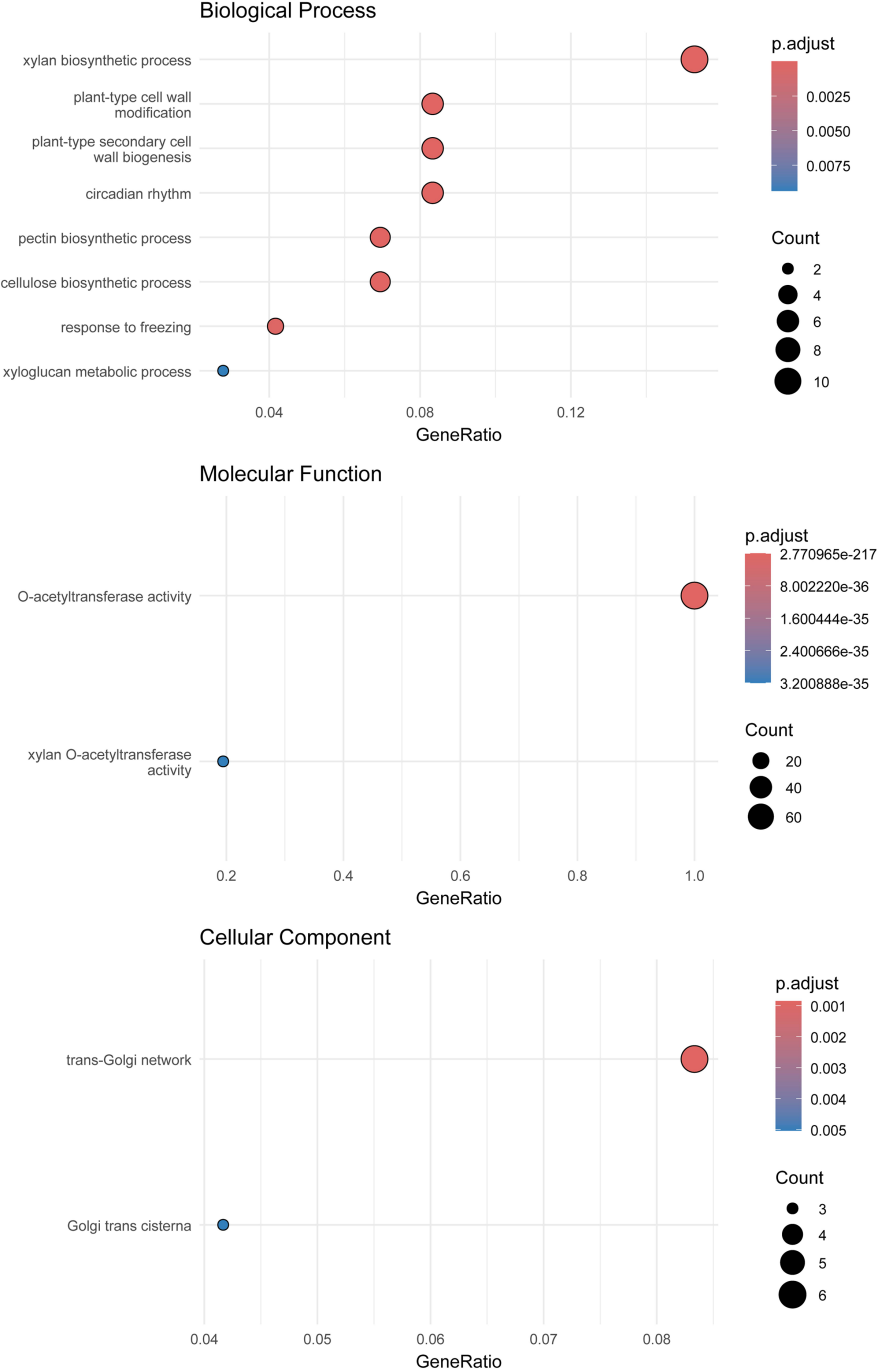
Gene Ontology analysis of the *StTBL* genes. Bubble diameter scales with quantitative magnitude, while color saturation encodes adjusted p-value significance.

GO enrichment analysis revealed eight biological processes, two molecular fun-ctions, and two cellular components associated with *StTBL* genes (**Figure 8**; **Supplementary Table S6**). Enriched biological processes included xylan biosynthetic process (11), plant-type cell wall modification (6), plant-type secondary cell wall biogenesis (6), pectin biosynthetic process (5), cellulose biosynthetic process (5), circadian rhythm (6), response to freezing (3), and xyloglucan metabolic process (2). Molecular functions terms were O-acetyltransferase activity (72) and xylan O-acetyltransferase activity (14). Cellular components included the trans-Golgi network (6) and Golgi trans cisterna (3). Functionally, *StTBL15*/*16*/*25*/*31*/*33*/*50*/*51*/*61*/*62*/*63*/*71* exhibited xylan O-acetyltransferase activity and were involved in xylan biosynthesis. Genes such as *StTBL2*/*64*/*65* were linked to cell wall modification and secondary wall biogenesis, while *StTBL2*/*50*/*62*/*64*/*65* participated in both pectin biosynthesis and cellulose biosynthesis. Cold stress-related processes included *StTBL25*, *StTBL31*, and S*tTBL33*. Localization analysis showed that *StTBL/38*/*40*/64/*65*/*70* were enriched in the trans-Golgi network, with *StTBL38*/*40*/*70* also enriched in the Golgi trans cisterna (**Supplementary Table S6**).

**Figure 9:**
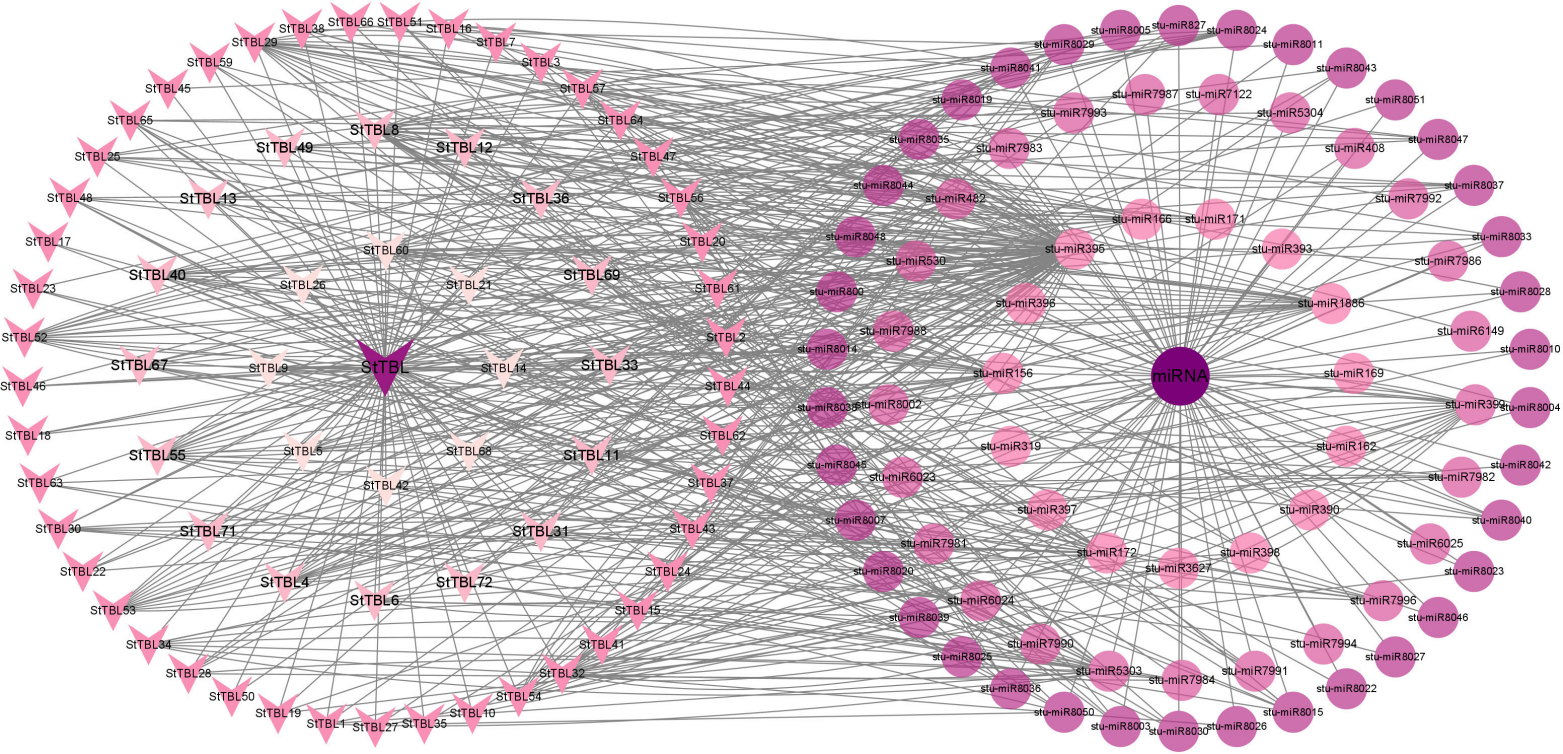
The interaction network of miRNA-StTBL. *StTBL* gene paralogs are encoded by the leftward arrow, and the circular representing distinct miRNA families.

77 putative miRNAs targeting 72 *StTBLs* genes were identified, while no miRNA targeted genes *StTBL39*/*58*/*70* (**Figure 9**). Among them, 90 stu-miR395 family members targeted 9 *StTBL* genes, 20 stu-miR5303 family members targeted 10 *StTBL* genes, 19 stu-miR156 family members targeted 7 *StTBL* genes, 16 stu-miR172 family members targeted 9 *StTBL* genes, 17 stu-miR1886 family members targeted 10 *StTBL* genes, 16 stu-miR399 family members targeted *StTBL25*/*32*/*41*, 11 stu-miR482 family members targeted 10 *StTBL* genes (**Supplementary Table S7**).

### 3.6 *StTBL* expression in potato tissues

**Figure 10:**
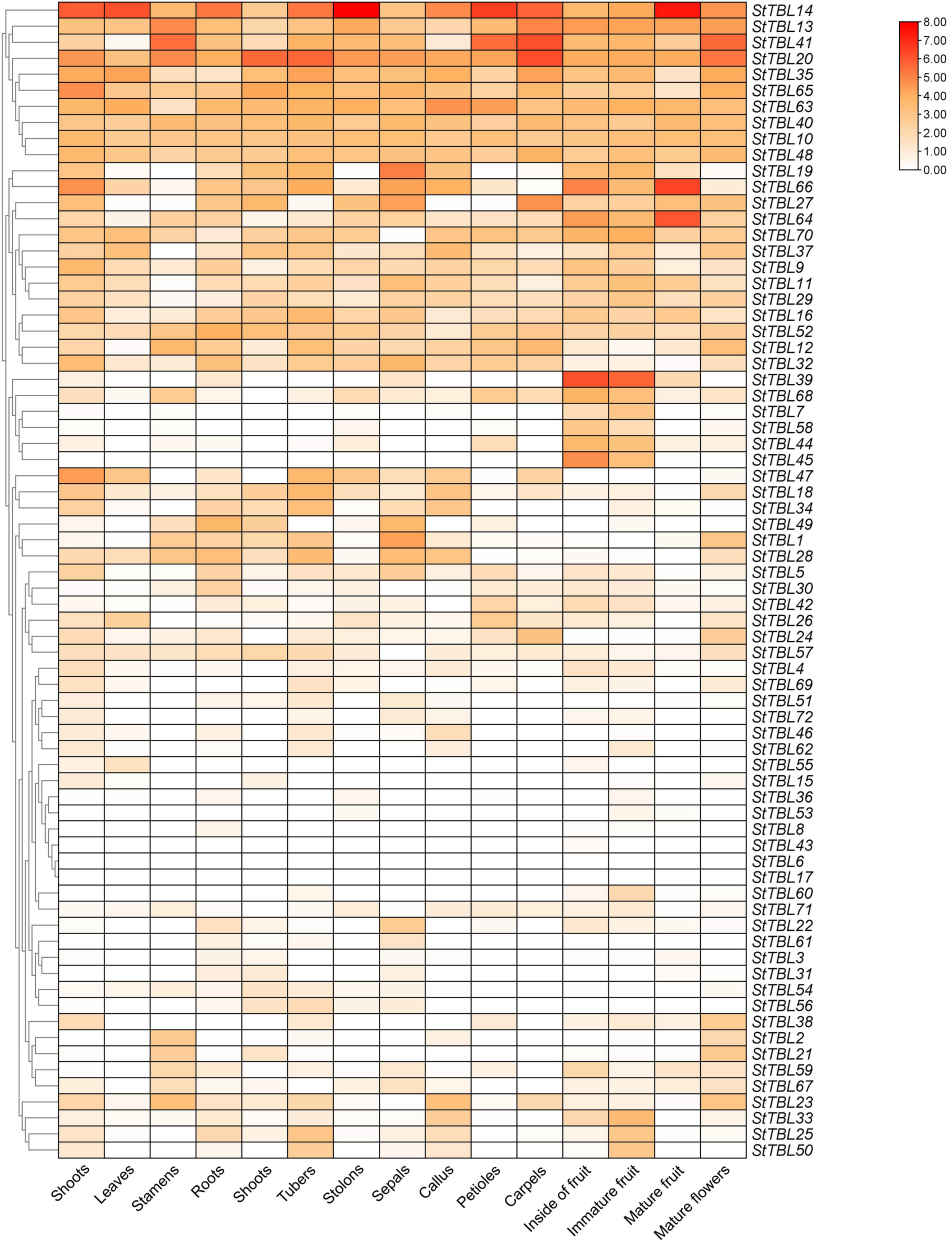
Differentially expressed St*TBL* genes over tissues in *S. tuberosum*.

To explore *StTBLs* gene expression across potato tissues, RNA-Seq transcriptome data were analyzed. The results showed that *StTBL9*/*10*/*11*/*12*/*13*/*14*/*16*/*18*/*20*/*32*/*35*/*40*/*41*/*48*/*52*/*63*/*64*/*48*/*63*/*64*/*65*/*70* were expressed in all tissues, with *StTBL14*/*20*/*40*/*48*/*63*/*64*/*65* showing consistently high expression. Specifically, *StTBL14* was the most highly expressed in shoots, leaves, sepals, carpels, petals, and mature fruit (FPKM > 5); *StTBL20* in tubers, stolons, petals, and mature fruit (FPKM > 5); and *StTBL64* and *StTBL66* in mature fruit (FPKM > 6). By contrast, *StTBL3*/*6*/*8*/*43*/*53* were either undetected or expressed at very low levels (FPKM < 1) (**Figure 10**; **Supplementary Table S8**). The clustering results revealed multiple pairs of *StTBL* genes sharing identical expression patterns. These included: *StTBL31* and *StTBL41*; *StTBL35* and *StTBL65*; *StTBL10* and *StTBL48*; *StTBL19* and *StTBL66*; *StTBL27* and *StTBL64*; *StTBL11* and *StTBL29*; *StTBL12* and *StTBL32*; *StTBL7* and *StTBL58*; *StTBL44* and *StTBL45*; *StTBL18* and *StTBL34*; *StTBL1* and *StTBL28*; *StTBL30* and *StTBL42*; *StTBL24* and *StTBL57*; *StTBL4* and *StTBL69*; *StTBL46* and *StTBL62*; *StTBL36* and *StTBL53*; and *StTBL51* and *StTBL72*. Additionally, *StTBL33*, *StTBL25*, and *StTBL50* also exhibited the same expression pattern.

### 3.7 Expression of *StTBL*s in potato under salt and drought treatment

**Figure 11:**
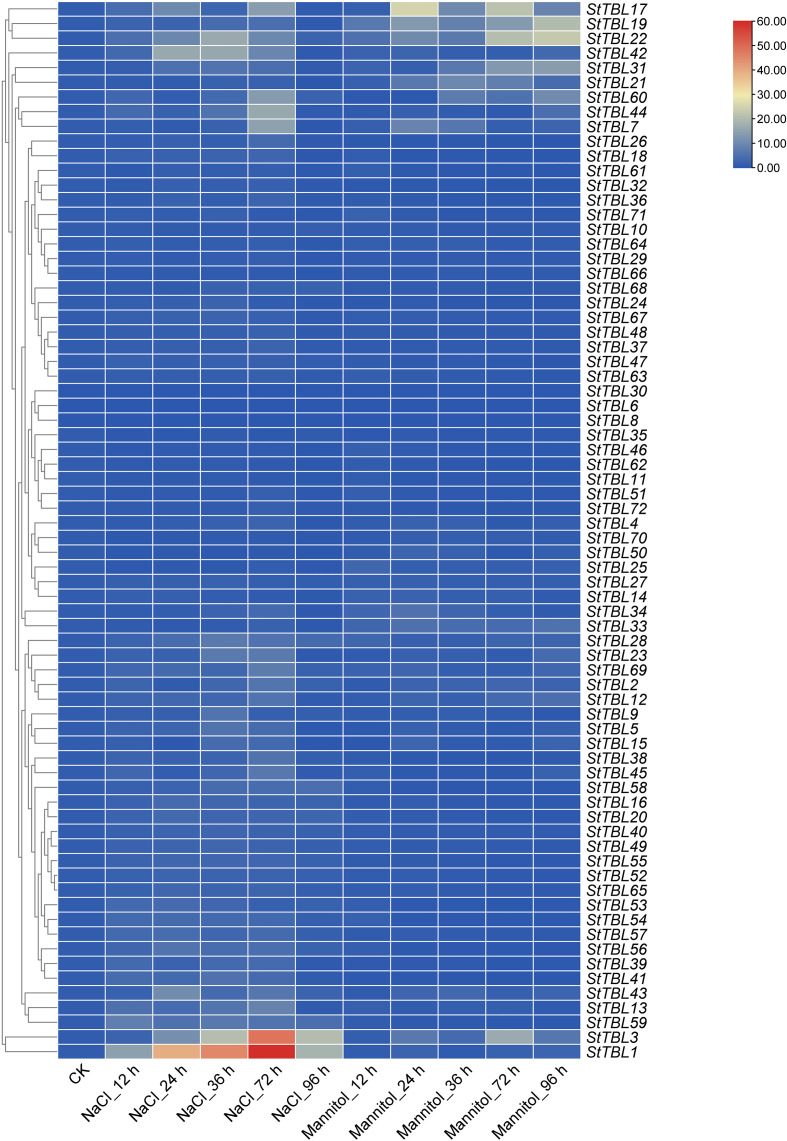
The gene expression levels of 72 *StTBL* genes under salt stress and drought stress at 0–96 h, the horizontal axis represents the treatment conditions, and the vertical axis represents the gene.

To evaluate the role of *StTBL* gene in abiotic stress response, qRT-PCR was used to analyze the gene expression of *StTBL* gene in leaves under salt stress and drought stress. Under NaCl stress, 57 *StTBL* genes showed significant expression changes rela-tive to the control, while 15 genes remained unchanged. *StTBL1*/*3*/*16*/*20*/*22*/*28*/*58/59*/*60*/*68* were consistently induced, and StTBL31/42/43/56 were induced during the early stage. *StTBL*15/22/28/41 reached peak expression at 36 h, whereas *StTBL1*/*3*/*7*/*13*/*19*/*44*/*60*/*69* peaked at 72 h. *StTBL68* was only induced at the late stage. These genes may function as positive regulators of salt stress responses. In contrast, *StTBL10*/*11*/*25*/*33*/*46*/*50*/*51*/*62*/*66*/*70*/*72* was down-regulated (**Figure 11**; **Supplementary Table 10**). Overall, these findings indicate that many *TBL* genes participate in salt stress response, with individual members exhibiting distinct regulatory patterns.

Under drought stress, *StTBL1/2/3/12/19/21/22/28/31/33/69* were significantly un-regulated, while *StTBL4/13/14/50/70* showed no significant change. *StTBL7/28/34* was induced early, and *StTBL43/44* were induced late. *StTBL21* exhibited the highest ex-pression at 36 h. In contrast, 43 genes (*StTBL5*/*9*/*10*/*11*/*15*/*16*/*18*/*20*/*24*/*26*/*29*/*32*/*35*/*36*/*37*/*38*/*39*/*40*/*41*/*45*/*46*/*47*/*48*/*49*/*51*/*52*/*53*/*54*/*55*/*56*/*57*/*58*/*59*/*61*/*62*/*63*/*64*/*65*/*66*/*67*/*68*/*71*/*72*) were down-regulated, showing a strong synergistic repression under drought stress (**Figure 11**). This suggests that many family members may contribute to stress sensitivity or to the function of metabolic or developmental pathways influenced by osmotic stress. Across both treatments, *StTBL6*/*8*/*30* showed no detectable expression, probably due to extremely low transcript abundance in leaves (0, 0, and 0.14, respec-tively; **Supplementary Table 8**). Cluster analysis revealed several groups with similar expression dynamics, including *StTBL1/3/59*, *StTBL16/20/58*, *StTBL19*/*22*, *StTBL28*/*33*, *StTBL60/21/31*, and *StTBL2/12/69*. *StTBL68* did not cluster with other stress-responsive genes.

### 3.8 Homology analysis of StTBL and AtTBL proteins

Protein sequence alignment revealed seven gene pairs with > 70% homology. Among them, AtTBL33–StTBL34 showed 78.261% identity (**Supplementary Figure 3**), followed by AtTBL29 and StTBL25 at 72.016% (**Supplementary Figure 4**), AtTBL33 and StTBL26 at 71.649% (**Supplementary Figure 5**), AtTBL28 and StTBL31 at 71.591% (**Supplementary Figure 6**), and AtTBL13 and StTBL11 at 71.023% (**Supplementary Figure 7**).

## 4 Discussion

The *TBL* gene family plays a central role in the O-acetylation of plant cell wall polysaccharides, a modification essential for proper cell wall formation, plant growth, and responses to biotic and abiotic stresses (Gao et al., 2017; Xin et al., 2007; Zhao et al., 2021). However, a genome-wide analysis of the *StTBL* gene family in potato has not previously been conducted. In this study, we systematically analyzed the *StTBL* gene family using bioinformatics approaches. Temporal expression profiling under drought and salt treatments (0–96 h) identified 10 salt-responsive candidates and 11 drought-responsive candidates.

*TBL* genes represent the major plant enzyme family responsible for polysaccharide O-acetylation (Anantharaman and Aravind, 2010; Lunin et al., 2020). Comparative genomic studies have revealed varying numbers of *TBL* genes across species: 50 in Rose (*Rosa chinensis*) (Tian et al., 2021), 65 in *Pyrus bretschneider* (Ban et al., 2025), 64 in *Populus trichocarpa* (Zhong et al., 2018c), 37 in *Dendrobium officinale* (Si et al., 2022), 46 in *A. thaliana* (Bischoff et al., 2010a), 66 in *O. sativa* (Gao et al., 2017), 69 in *S. lycopersicum* (Zhong et al., 2020), 130 in *Nicotiana tabacum* (Wang et al., 2025), 49 in *Eucalyptus grandis* (Tang et al., 2024), 131 in *Gossypium hirsutum*, 130 in *Gossypium barbadense* (Zhu et al., 2024), and 37 in *Dendrobium officinale* (Si et al., 2022). Among these, *A. thaliana* has been the most extensively studied: of its 46 *TBL* genes, *AtTBL3*/*28*/*29/30*, *AtTBL31*/*32*/*33*/*34*/*35* are associated with xylan (Xiong et al., 2013; Yuan et al., 2016a, Yuan et al., 2016c, Yuan et al., 2016b; Zhong et al., 2017), *AtTBL19*/*20*/*21*/*22/27* with xyloglucan (Zhong et al., 2020) (Gille et al., 2011; Zhong et al., 2020; Zhu et al., 2014), *AtTBL23/24*/*25*/*26* with mannan (Zhong et al., 2018b), and *AtTBL46/TBR* with pectin (Bischoff et al., 2010a). Our study identified 72 *TBL* genes in potato, a substantially higher number than in *A. thaliana*. This expansion likely reflects evolutionary replication events, including possible whole-genome duplication.

The *TBL* genes of *S. tuberosum* and *A. thaliana* are only separated by 3–5 genes on average, highlights the evolutionary conservation of this family among dicotyle-donous plants. The *StTBL* gene in potato is orthologous to the *A. thaliana* Group II *TBL28–35* acetylation-related subfamily. It may have similar molecular functions, such as participating in acetylation modification. Collinearity analysis revealed stronger synteny among dicotyledonous plants compared with monocots, reflecting closer phylogenetic relationships. Chromosomal mapping revealed an uneven distribution, suggesting region-specific amplification. Moreover, all duplicated gene pairs exhibited Ka/Ks < 1, indicating strong purifying selection and functional conservation of *StTBL* genes during evolution, consistent with the results in *R. chinensis* (Tian et al., 2021), *N. tabacum* (Wang et al., 2025), and *G. hirsutum* (Zhu et al., 2024).

Length variation in StTBL proteins reflects functional diversity within the family, with their isoelectric point ranges suggesting heterogeneity that could affect protein-protein interactions and subcellular localization. Motifs 1–10 are essential for core biochemical activity (likely acetylation) and are evolutionarily conserved. All three subgroups contain GCD (motif 1), DXXH (motif 2), and motif 4, as also observed in *N. tabacum* (Wang et al., 2025). Many *StTBL* genes possess both PC-esterase and PMR5N domains, similar to findings in *R. chinensis* (Tian et al., 2021). Notably, *StTBL33*/*49*/*60* may have roles beyond cell wall modification; *StTBL33*, for instance, could be involved in regulating cell wall formation, cellulose, and xylan synthesis. Sixteen genes contain the conserved PLN02629 domain, reported also in *G. hirsutum*, *N. tabacum*, and *P. bretschneideri*, indicating its potential importance. Group I, with frequent PC-Esterase and PMR5N domains, represents a classical acetyltransferase branch. In Group II, PC-esterase is more conserved than PMR5N, pointing to functional divergence. Group III may originate from recent tandem duplications or retrotranspositions, with higher PMR5N content possibly indicating positive selection. This subgroup uniquely retains the GCD and DXXH motifs, associated with glycosylation and acid catalytic activity respectively, suggesting distinct substrate specificity (Wang et al., 2025). *StTBL33*/*49*/*60* belong to the PMR5N superfamily, while 16 genes are part of the PLN02629 superfamily, underscoring *TBL* multi-functionality in cell wall metabolism—a pattern also noted in *G. hirsutum*, *N. tabacum*, and *P. bretschneideri* (Ban et al., 2025; Wang et al., 2025; Zhu et al., 2024). Gene structure analysis shows considerable exon number variation (1–15). Single-exon genes like *StTBL39* may arise via retrotransposition, whereas multi-exon genes (e.g., *StTBL30* with 15 exons) could gain novel functions through exon shuffling. Fewer-exon genes likely perform core functions, while genes with more exons may undergo alternative splicing to produce isoforms responsive to environmental or endogenous signals (Lin et al., 2024).

GO analysis revealed that all 72 genes participate in cell wall xylan O-acetylation, with the initial acetylation step likely occurring in the Golgi apparatus (Manabe et al., 2011). Specifically, *StTBL2*/*38*/*40*/*64*/*65*/*70* may function in the initiation of xylan backbone acetylation, while *StTBL25*, *StTBL31*, and *StTBL33* may be involved in cold stress responses. In addition, cis-acting elements in *StTBL* promoters also may play a pivotal role in regulating gene expression related to growth, hormonal signaling, and stress responses. *StTBL* genes are involved in abiotic stress responses (such as drought and salt) and hormone signaling pathways.

These StTBL-targeting miRNAs may integrate multiple signals related to potato growth, development, and stress responses. Specifically, developmental cues—such as stu-miR156/172-regulated morphogenesis, tuberization, and flowering time—and nutrition/stress signals—including miR395/miR399-mediated sulfur/phosphorus meta-bolism and salt adaptation—could converge on the regulation of specific *StTBL* genes (He et al., 2024; Luo et al., 2024; Pegler et al., 2020; Yang et al., 2022). As TBL proteins directly participate in modifications like cell wall acetylation, changes in their expression may finely tune the wall’s mechanical and chemical properties, positioning them as key hubs linking internal signaling with external morphogenesis or biotic/abiotic stress resistance. For example, during pathogen infection, stu-miR482-mediated regulation of NBS-LRR immune genes may occur alongside its targeting of certain *StTBL* genes (Luo et al., 2023), potentially enabling a dual defense strategy that combines classical immunity with rapid adjustment of cell wall barriers.

*StTBL9*/*10*/*11*/*12*/*13*/*14*/*16*/*18*/*20*/*32*/*35*/*40*/*41*/*48*/*52*/*63*/*64*/*48*/*63*/*64*/*65*/*70* likely participate in fundamental physiological processes and function. Among these, *StTBL14*/*20*/*40*/*48*/*63*/*64*/*65* may play a role in fundamental physiological processes. *StTBL14* may play an important regulatory role in both vegetative growth and reproductive development, while *StTBL20* may contribute to storage organ formation and reproductive development.

*StTBL1* and *StTBL3* may have a synergistic effect in response to salt and drought stress. *StTBL1*, *StTBL3* and *StTBL59*, *StTBL16*, *StTBL20* and *StTBL5* may synergistic involvement in salt tolerance. Several gene pairs or groups (such as *StTBL19*/*22*, *StTBL21*/*31*, *StTBL28*/*33*, and *StTBL69*/*2*/*12*) indicate that they may function together in coordinated pathways to respond to drought. It is worth noting that both *StTBL2* and *StTBL12* contain MBS elements, which further provides a basis for their potential drought tolerance. Notably, the GO annotation for *StTBL2* indicates a possible role in cell wall formation, supporting its involvement in stress-associated structural regulation. In addition, *StTBL3* and *StTBL69* also contained MBS elements. These results further speculated that *StTBL3* responded to salt and drought stress and the potential drought tolerance of *StTBL69*. In addition, the tissue expression patterns were similar between *StTBL1* and *StTBL28*; *StTBL3* and *StTBL31*; and *StTBL2* and *StTBL21*. This suggests that the genes within each pair may function together in the same biological pathway or as part of a complex.

StTBL31, like AtTBL28, may be a key xylan o-acetyltransferase involved in plant cell wall biosynthesis. *StTBL31* showed a sustained increase in expression under drought stress. Together with its high sequence homology and collinearity with *AtTBL28*, as well as GO annotations indicating involvement in xylan synthesis and cold-stress responses, these findings suggest that *StTBL31* participates in long-term protective responses rather than transient stress reactions. StTBL31 may function as a conserved xylan acetyltransferase induced by osmotic stress, contributing to drought and environmental adaptation by dynamically modifying cell wall xylans. This makes *StTBL31* a strong candidate gene for molecular breeding of drought-resistant potato, with potential roles extending beyond cell wall synthesis to include integrating stress signaling and cell wall integrity pathways. No collinearity between the potato *TBL* gene and *AtTBL33*, although StTBL34 and StTBL26 proteins share high homology with AtTBL33. This suggests conserved functional evolution, while gene duplication and functional diversification in potato may enhance regulatory complexity despite loss of positional correspondence.

Collectively, this study shows that *StTBL* genes play central roles in cell wall modification, chloroplast function, and stress responses. These findings provide valuable candidate genes for improving potato stress resistance.

## 5 Conclusions

This study comprehensively characterized 72 *TBL* genes in potato, delineating their conserved PC-esterase domain. Phylogenetic analysis divided these genes into three groups based on distinct structures and motif compositions, while chromosomal mapping revealed an uneven distribution of *TBL* genes across the 12 potato chrom-osomes. Tandem and fragment duplication events were identified as key evolutionary forces driving the *StTBL* family expansion. Collinearity analysis with six representative species further provided insights into evolutionary conservation, offering a basis for future comparative functional studies. Expression profiling revealed tissue-specific patterns and dynamic responses of 72 genes under drought and salt stress. 10 genes (*StTBL1*/*3*/*16*/*20*/*22*/*28*/*58*/*59*/*60/68*) were identified as potential salt-responsive cand-idates, while 11 genes (*StTBL1*/*2*/*3*/*12*/*19*/*21*/*22*/*28*/*31*/*33*/*69*) were potential drought-responsive candidates. Notably, *StTBL1*/*3*/*22*/*28* responded to both salt and drought stress. In addition, *StTBL1*/*2*/*16*/*22*/*31*/*33* are likely involved in cell wall formation or modification. Collectively, these findings identify key *StTBL* genes as promising targets for further functional validation and the development of stress-resilient potato cultivars.

## Data Availability

The original contributions presented in the study are included in the article/**Supplementary Material**. Further inquiries can be directed to the corresponding author.
