# Supplementary material for: Comprehensive characterization of potato TBL genes reveals candidates for salt and drought stress tolerance
Source: Front Plant Sci. 2026 Jan 26;16:1741231. doi: 10.3389/fpls.2025.1741231 (PMC12884326; doi:10.3389/fpls.2025.1741231)
Supplement: Supplementary file 11 [file DataSheet1.docx]

**Supplementary materials**


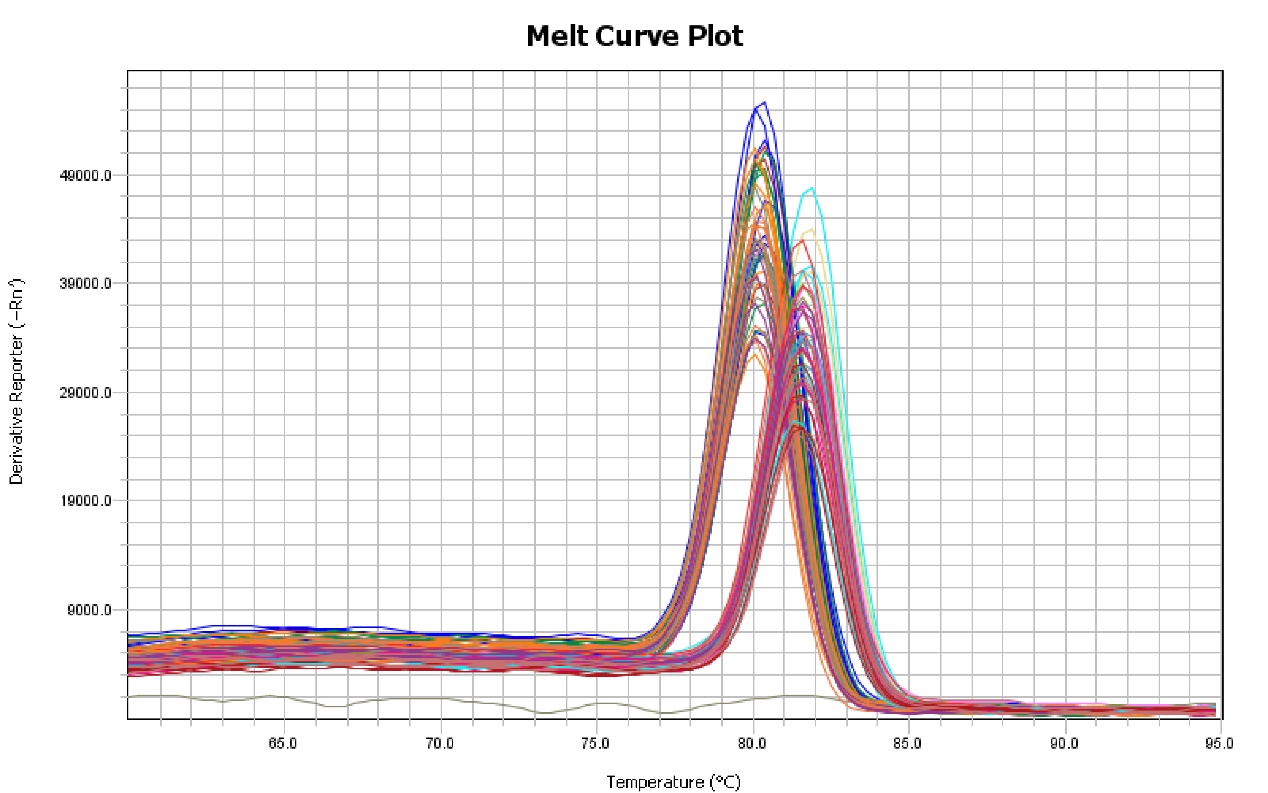


**Figure S1**. qRT-PCR melting curve of transgenic plants


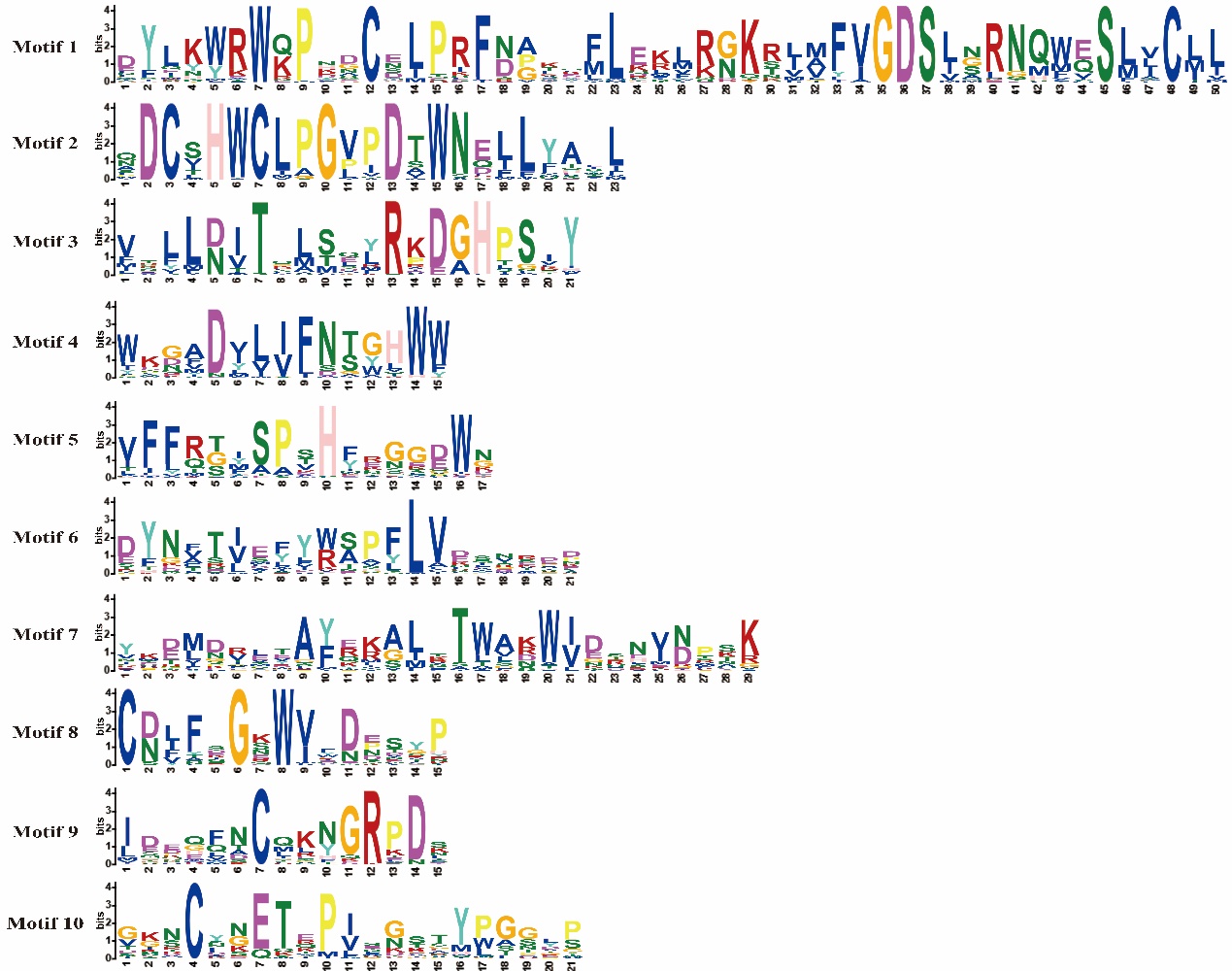


**Figure S2**. Putative motifs of the StTBL proteins in foxtail millet

**Figure S3**. Protein sequence alignment of AtTBL33 and StTBL34

**Figure S4**. Protein sequence alignment of AtTBL29 and StTBL25

**Fig****ure S5**. Protein sequence alignment of AtTBL33 and StTBL26

**Figure S6**. Protein sequence alignment of AtTBL28 and StTBL31

**Figure S7**. Protein sequence alignment of AtTBL13 and StTBL11
